# Antibacterial Activity of Venom from the Puff Adder (*Bitis arietans*), Egyptian Cobra (*Naja haje*), and Red Spitting Cobra (*Naja pallida*)

**DOI:** 10.1155/2023/7924853

**Published:** 2023-03-02

**Authors:** Mitchel Otieno Okumu, Kennedy Lojau Eyaan, Luke Kipkorir Bett, Nduhiu Gitahi

**Affiliations:** ^1^Department of Public Health, Pharmacology and Toxicology, Faculty of Veterinary Medicine, University of Nairobi, P.O. Box 29053-00625, Nairobi, Kenya; ^2^Department of Health, County Government of Kisumu, P.O. Box 2738-40100, Kisumu, Kenya

## Abstract

*Bitis arietans* (Puff adder), *Naja haje* (Egyptian cobra), and *Naja pallida* (Red spitting cobra) venoms were tested for antimicrobial activity. This evaluation employed disc diffusion and microbroth dilution techniques. Gram-positive bacteria (*Bacillus cereus* and *Staphylococcus aureus*) and Gram-negative bacteria (*Escherichia coli*, *Klebsiella pneumonia*, and *Salmonella typhi*) were used. Aztreonam (30 *µ*g), cefpodoxime (10 *µ*g), cefoxitine (30 *µ*g), streptomycin (25 *µ*g), ceftriaxone (30 *µ*g), nalidixic acid (30 *µ*g), tetracycline (30 *µ*g), and sulfamethoxazole (25 *µ*g) were used as controls. All tests were conducted in triplicate (*n* = 3). *Results*. The activity of *B. arietans* venom against Gram-negative bacteria was significantly lower (*p* < 0.001) than that of controls. The efficacy of *B. arietans* venom and sulfamethoxazole against both Gram-positive and Gram-negative bacteria was not significantly different (*p* > 0.9999). The efficacy of *B. arietans* venom against Gram-positive bacteria was significantly lower (*p* < 0.001) than cefoxitin, streptomycin, and tetracycline. The efficacy of *N. haje* venom against Gram-negative bacteria was significantly lower (*p* < 0.001) than that of controls. There was no significant difference in the antimicrobial efficacy of *N. haje* venom and controls against Gram-positive bacteria (*p*=0.3927 to *p*=0.9998). There was no significant difference in the efficacy of *N. pallida* venom and controls against Gram-negative bacteria (*p*=0.3061 to *p*=0.9981). There was no significant difference in the efficacy of *N. pallida* venom and controls against Gram-positive bacteria (*p*=0.2368 to *p* > 0.9999). *Conclusions*. Of all the tested venoms, only *Naja pallida* venom showed good efficacy against both Gram-positive and Gram-negative bacteria.

## 1. Background

*Bacillus cereus, Staphylococcus aureus*, *Escherichia coli,* and *Klebsiella pneumoniae* are some of the pathogens of medical importance in the developing World [1–4]. These pathogens have been implicated in food poisoning [5, 6], sepsis [7], and neonatal infections [8, 9]. Reports on drug resistance for clinical isolates of these pathogens are rife in the scientific literature [10–12]. Therefore, new and innovative therapies are urgently required to mitigate the unfolding AMR crisis. Venom from animals comprises a complex cocktail of pharmacological molecules that could help bolster the ranks of the current antimicrobial agents. *Bitis arietans* (puff adder, a viper)*, Naja haje* (Egyptian cobra, a nonspitting cobra), and *Naja pallida* (Red spitting cobra, a spitting cobra) are snakes of medical importance in Subsaharan Africa [13]. Venom from these snakes could be useful in combating medically important pathogens in the region including *Bacillus cereus, Staphylococcus aureus*, *Escherichia coli,* and *Klebsiella pneumoniae.* However, data to support their efficacy against common bacterial pathogens are not available. The aim of the present study was therefore to determine the antibacterial activities of venoms from *Bitis arietans*, *Naja haje*, and *Naja pallida* against *Bacillus cereus, Staphylococcus aureus*, *Escherichia coli,* and *Klebsiella pneumoniae.*

## 2. Materials and Methods

### 2.1. Venom

Venoms from *Bitis arietans* (Puff adder), *Naja haje* (Egyptian Cobra), and *Naja pallida* (Red spitting Cobra) were collected from wild-caught snakes at the East African Venom Supplies (Kenya). They were lyophilized and stored at −4°C at the Pharmacology and Toxicology Lab, Faculty of Veterinary Medicine, University of Nairobi. Each of the lyophilized venoms were accurately weighed (0.25 g) in an analytical balance and triturated using a pestle and mortar. The triturated venoms were transferred to 10 mL volumetric flasks and made up to the mark with phosphate buffered saline to make a 25 mg/mL concentration.

### 2.2. Microbial Cultures

Standard microbial cultures of *E. coli* ATCC 25922, *K. pneumoniae* ATCC 700603, *S. aureus* ATCC 25923, *B. cereus* ATCC 11778, and *S. typhi* ATCC 6539 were obtained from the Research Unit of the Bacteriology Lab at the Faculty of Veterinary Medicine, University of Nairobi. All the bacteria were subcultured in Mueller Hinton Agar (MHA) for susceptibility testing after 24 hours.

### 2.3. Antimicrobial Discs

Antimicrobial discs were prepared by punching Whatman Filter paper number 1 using an office paper punch. The prepared discs were sterilized by autoclaving at 121°C for 15 minutes. The discs were then soaked for 5 minutes in the prepared venom concentrations (25 mg/mL) using Petri dishes. The venom-soaked discs were then gently picked using forceps and dried in an oven at 37°C for 30 minutes.

### 2.4. Growth Media

Mueller Hinton Agar (MHA) was prepared by dissolving 19 g of the media in distilled water, heating in an oven to boil and dissolve for 5 minutes. This was followed by sterilization via an autoclave at 121°C for 15 minutes. The medium was allowed to cool in a 45°C water bath, poured into plates under the sterile laminar flow hood, and left to solidify for 10 minutes. The prepared Mueller Hinton Agar (MHA) plates were transferred to an oven and allowed to dry at an angle of 45°C for 10 minutes.

### 2.5. Microbial Inoculum

0.85% normal saline was placed in the same tubes as used in the McFarland standard and sterilized by autoclaving at 121°C for 15 minutes. The inoculum of the microorganisms was prepared by suspending isolated bacterial colonies from the pure microbial subcultures in 0.85% normal saline, and the turbidities were standardized to 0.5 McFarland units.

### 2.6. Antimicrobial Activity Assay

The disk diffusion test and minimum inhibitory concentration (MIC) determination were used to screen for antibacterial activity according to standard techniques described in previous publications [14–16]. In brief, the bacterial strains were grown in trypticase soy agar at 37°C for 16–18 hours before being adjusted to 0.5 McFarland standards (1.5 × 10^8^ colony forming unit/mL) with sterile normal saline [14–16]. A sterile swab was dipped into the inoculum, and excess inoculums were removed by pressing firmly against the side of the tube above the liquid level. The swab was streaked three times across the surface of Mueller−Hinton agar plates [14–16]. Each snake venom solution was prepared at a concentration of 25 mg/mL by dissolving it in sterile deionized water. 1 *µ*L solutions of the prepared venom concentration were applied to Whatman paper discs (6 mm diameter) and placed on bacterial culture in the triplicate assay, which was then incubated for 16–24 hours at 35 ± 2°C [14–16]. The zones of inhibition around the discs were measured using a digital Vernier caliper. Distilled water discs were used as the negative control. Positive controls included aztreonam (30 *µ*g), cefpodoxime (10 *µ*g), cefoxitin (30 *µ*g), streptomycin (25 *µ*g), ceftriaxone (30 *µ*g), nalidixic acid (30 *µ*g), tetracycline (30 *µ*g), and sulfamethoxazole (25 *µ*g). The snake venoms with the largest inhibition zone diameters were chosen for minimum inhibitory concentration (MIC) determination [14–16]. The Clinical Laboratory Standards Institute (CLSI 2014) broth microdilution method was used for the MIC test [14–16]. The culture was diluted to 10^6^ CFU/mL after 3 hours of the bacterial growth. The snake venom was diluted twice with Mueller−Hinton broth, and then the diluted bacterial culture was added to achieve a final concentration ranging from 0.39 mg/mL to 25.0 mg/mL. After 16–18 hours of incubation at 35 ± 2°C, the MIC was defined as the lowest concentration of venom or antibiotic preventing the visible bacterial growth when compared to the positive growth control (medium plus bacteria without venom or antibiotic) with high turbidity and to the negative growth control (medium plus bacteria without venom or antibiotic) [14–16].

### 2.7. Data Analysis

Data on the zones of inhibition of conventional antibiotics and venoms against Gram-positive and Gram-negative bacteria were summarized on MS Excel 2016 spreadsheet and imported into GraphPad Prism. Two-Way Analysis of Variance (ANOVA) and Dunnet's post hoc test were then performed with *p* < 0.05 considered significant.

## 3. Results

**Figure 1:**
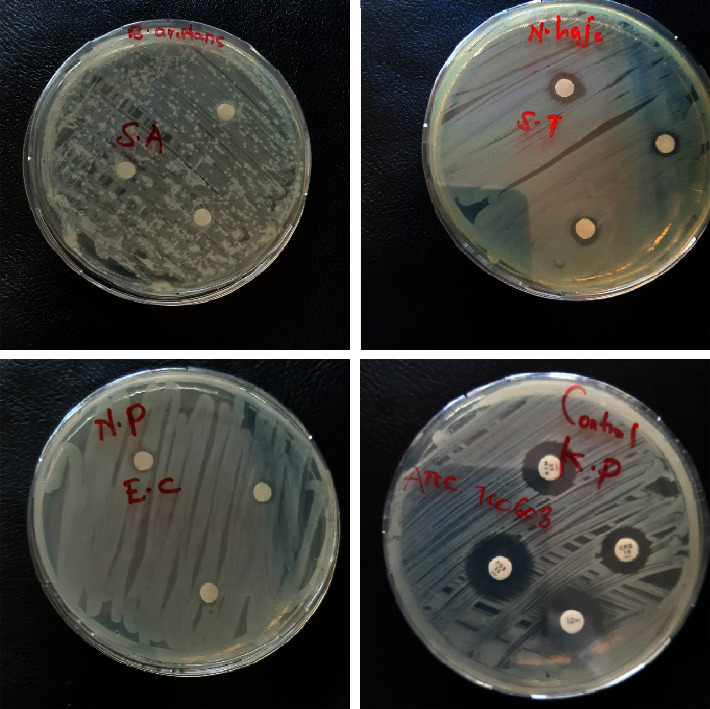
Illustration of the zones of inhibition produced by conventional antibiotics and venoms on selected bacteria. SA: *Staphylococcus aureus*, ST: *Salmonella typhi*, NP: *Naja pallida*, EC: *Escherichia coli*, KP: *Klebsiella pneumoniae* (*n* = 3).

Figure 1 illustrates the effect of conventional antibiotics and venoms on selected bacteria.  Table 1 shows the mean size of the clearing zones or zones of inhibition values of venoms and conventional antibiotics against Gram-positive and Gram-negative bacteria.

The effect of the conventional antibiotics and venoms on *E. coli* was in the order ATM > CRO > TCY > CPD > CXT > NAL > SMZ > STM > NHV > NPV > BAV as shown in Table 1. The effect of the conventional antibiotics and venoms on *K. pneumoniae* was in the order TCY > CRO > CXT > NAL > STM > ATM > CPD > NHV > NPV > SMZ∼BAV as shown in Table 1. The effect of the conventional antibiotics and venoms on *S. aureus* was in the order STM > TCY > CRO > CXT > NHV > NPV > ATM∼CPD∼NAL∼SMZ∼BAV as shown in Table 1. The effect of the conventional antibiotics and venoms on *B. cereus* was in the order STM > TCY > CXT > CRO > NPV > NHV > ATM∼CPD∼NAL∼BAV as shown in Table 1. The effect of the conventional antibiotics and venoms on *S. typhi* was in the order CRO > CXT > STM > TCY > NHV > NPV > ATM∼NAL∼SMZ∼BAV as shown in Table 1.

**Figure 2:**
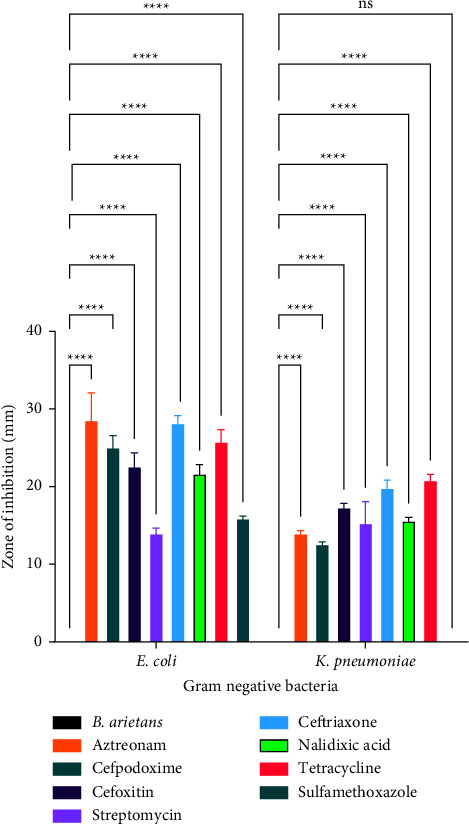
A comparison of the antibacterial effect of *Bitis arietans* venom and conventional antibacterial agents against gram negative bacteria.

Figure 2 is a comparison of the antibacterial effect of venom from *Bitis arietans* venom and conventional antibacterial agents against Gram-negative bacteria. The effect of *B. arietans* venom on *E. coli* was significantly lower (*p* < 0.001) than the effect of cefpodoxime, cefoxitin, streptomycin, ceftriaxone, nalidixic acid, tetracycline, and sulfamethoxazole as shown in Figure 2. The effect of *B. arietans* venom on *K. pneumoniae* was significantly lower (*p* < 0.001)than the effect of cefpodoxime, cefoxitin, streptomycin, ceftriaxone, nalidixic acid, tetracycline, and sulfamethoxazole as shown in Figure 2. There was no significant difference (*p* > 0.9999) in the effect of *Bitis arietans* venom and Sulfamethoxazole on *K. pneumoniae* as shown in Figure 2.

**Figure 3:**
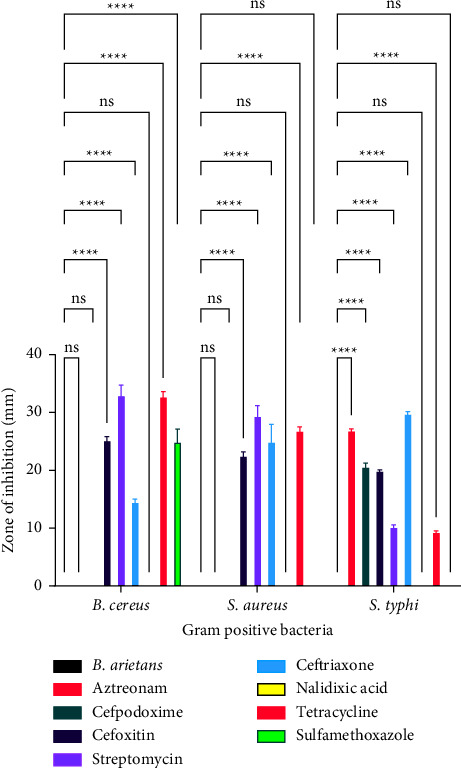
A comparison of the antibacterial effect of *Bitis arietans* venom and conventional antimicrobial agents against gram positive bacteria.

Figure 3 shows a comparison of the antibacterial effect of *Bitis arietans* venom and conventional antimicrobial agents against Gram-positive bacteria. The effect of *B. arietans* venom on *S. aureus* was significantly lower (*p* < 0.001) than the effect of cefoxitin, streptomycin, ceftriaxone, and tetracycline as shown in Figure 3. There was no significant difference (*p* > 0.9999) in the effect of *Bitis arietans* venom, aztreonam, cefpodoxime, nalidixic acid, and sulfamethoxazole on *S. aureus* as shown in Figure 3. The effect of *B. arietans* venom on *S. typhi* was significantly lower (*p* < 0.001) than the effect of aztreonam, cefpodoxime, cefoxitin, streptomycin, ceftriaxone, and tetracycline as shown in Figure 3. There was no significant difference (*p* > 0.9999) in the effect of *Bitis arietans* venom, nalidixic acid, and sulfamethoxazole on *S. typhi* as shown in Figure 3.

**Figure 4:**
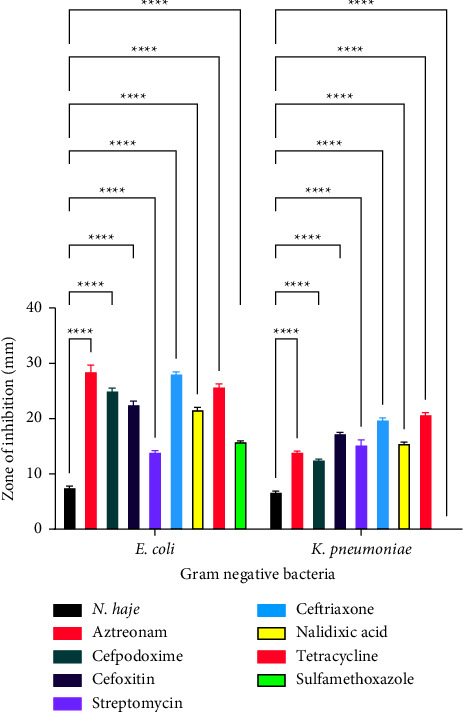
A comparison of the antibacterial effect of *Naja haje* venom and conventional antimicrobial agents against gram negative bacteria.

Figure 4 shows a comparison of the antibacterial effect of *Naja haje* venom and conventional antimicrobial agents against Gram-negative bacteria. The effect of *N. haje* venom on *E. coli* and *K. pneumoniae* was significantly lower (*p* < 0.001) than the effect of aztreonam, cefpodoxime, cefoxitin, ceftriaxone, nalidixic acid, tetracycline, and sulfamethoxazole as shown in Figure 4.

**Figure 5:**
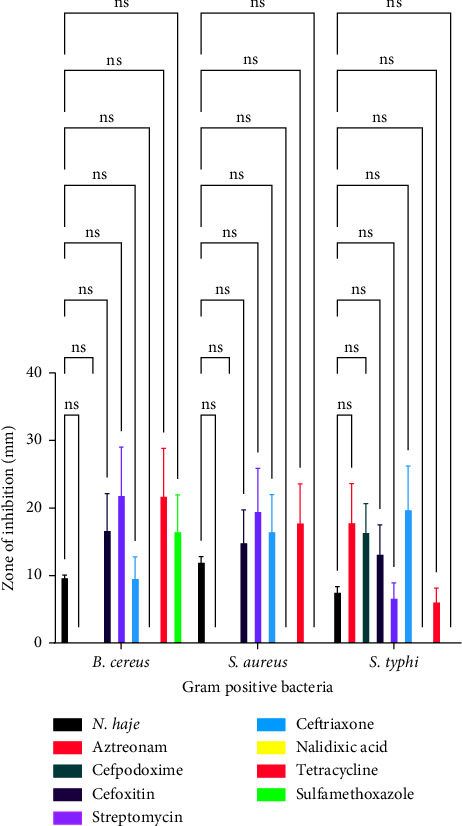
A comparison of the antibacterial effects of *Naja haje* venom and conventional antimicrobial agents against gram positive bacteria.

Figure 5 is a comparison of the antibacterial activity of *Naja haje* venom and conventional antimicrobial agents against Gram-positive bacteria. With regard to *B. cereus*, there was no significant difference between the effect of *Naja haje* venom and aztreonam (*p*=0.6857), *Naja haje* venom and cefpodoxime (*p*=0.7388), *Naja haje* venom and cefoxitin (*p*=0.9356), *Naja haje* venom and ceftriaxone (*p*=0.3927), *Naja haje* venom and nalidixic acid (*p*=0.6857), *Naja haje* venom and tetracycline (*p*=0.4081), and *Naja haje* venom and sulfamethoxazole (*p*=0.9445) as shown in Figure 5. With regard to *S. aureus*, there was no significant difference between the effect of *Naja haje* venom and aztreonam (*p*=0.4023), *Naja haje* venom and cefpodoxime (*p*=0.4699), *Naja haje* venom and cefoxitin (*p*=0.9998), *Naja haje* venom and ceftriaxone (*p*=0.9067), *Naja haje* venom and nalidixic acid (*p*=0.9958), *Naja haje* venom and tetracycline (*p*=0.4023), and *Naja haje* venom and sulfamethoxazole (*p*=0.9787) as shown in Figure 5. With regard to *S. typhi*, there was no significant difference between the effect of *Naja haje* venom and aztreonam (*p*=0.6349), *Naja haje* venom and cefpodoxime (*p*=0.8358), *Naja haje* venom and cefoxitin (*p*=0.9829), *Naja haje* venom and ceftriaxone (*p*=0.9999), *Naja haje* venom and nalidixic acid (*p*=0.3946), *Naja haje* venom and tetracycline (*p*=0.8931), and *Naja haje* venom and sulfamethoxazole (*p* > 0.9999) as shown in Figure 5.

**Figure 6:**
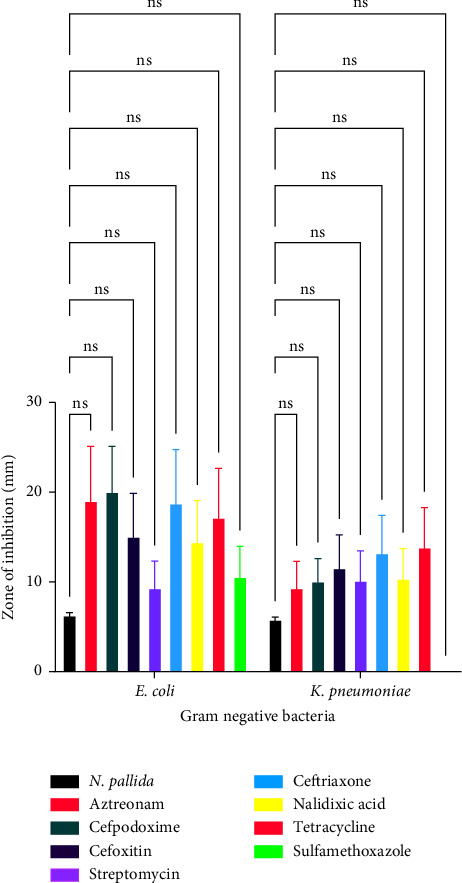
A comparison of the antibacterial effects of *Naja pallida* venom and conventional antimicrobial agents against gram negative bacteria.

Figure 6 is a comparison of the antibacterial effects of *Naja pallida* venom and conventional antimicrobial agents against Gram-negative bacteria. With regard to *E. coli*, there was no significant difference between the effect of *Naja pallida* venom and aztreonam (*p*=0.3439), *Naja pallida* venom and cefpodoxime (*p*=0.3061), *Naja pallida* venom and cefoxitin (*p*=0.8041), *Naja pallida* venom and ceftriaxone (*p*=0.3725), *Naja pallida* venom and nalidixic acid (*p*=0.8608), *Naja pallida* venom and tetracycline (*p*=0.5628), and *Naja pallida* venom and sulfamethoxazole (*p*=0.9971) as shown in Figure 6. With regard to *K. pneumoniae*, there was no significant difference between the effect of *Naja pallida* venom and aztreonam (*p*=0.9993), *Naja pallida* venom and Cefpodoxime (*p*=0.9981), *Naja pallida* venom and Cefoxitin (*p*=0.9804), *Naja pallida* venom and ceftriaxone (*p*=0.9967), *Naja pallida* venom and nalidixic acid (*p*=0.9144), *Naja pallida* venom and tetracycline (*p*=0.9955), and *Naja pallida* venom and sulfamethoxazole (*p*=0.8690) as shown in Figure 6.

**Figure 7:**
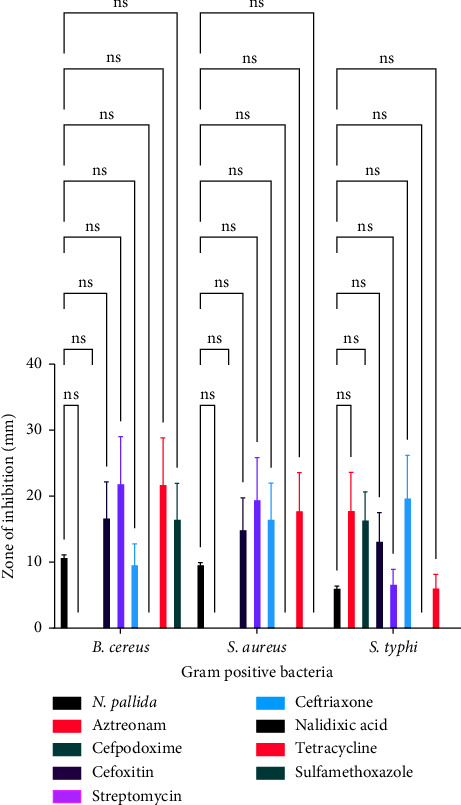
A comparison of the antibacterial effects of *Naja pallida* and conventional antimicrobial agents against gram positive bacteria.

Figure 7 is a comparison of the antibacterial effects of *Naja pallida* and conventional antimicrobial agents against Gram-positive bacteria. With regard to *B. cereus*, there was no significant difference between the effect of *Naja pallida* venom and aztreonam (*p*=0.5583), *Naja pallida* venom and cefpodoxime (*p*=0.6217), *Naja pallida* venom and cefoxitin (*p*=0.9741), *Naja pallida* venom and streptomycin (*p*=0.5142), *Naja pallida* venom and ceftriaxone (*p* > 0.9999), *Naja pallida* venom and nalidixic acid (*p*=0.5583), *Naja pallida* venom and tetracycline (*p*=0.5310), and *Naja pallida* venom and sulfamethoxazole (*p*=0.9786) as shown in Figure 7.

With regard to *S. aureus*, there was no significant difference between the effect of *Naja pallida* venom and aztreonam (*p*=0.6964), *Naja pallida* venom and cefpodoxime (*p*=0.7483), *Naja pallida* venom and cefoxitin (*p*=0.9882), *Naja pallida* venom and streptomycin (*p*=0.6744), *Naja pallida* venom and ceftriaxone (*p*=0.9386), *Naja pallida* venom and nalidixic acid (*p*=0.6964), *Naja pallida* venom and tetracycline (*p*=0.8535), and *Naja pallida* venom and sulfamethoxazole (*p*=0.6964) as shown in Figure 7.

With regard to *S. typhi*, there was no significant difference between the effect of *Naja pallida* venom and aztreonam (*p*=0.4423), *Naja pallida* venom and cefpodoxime (*p*=0.6779), *Naja pallida* venom and cefoxitin (*p*=0.9281), *Naja pallida* venom and streptomycin (*p* > 0.9999), *Naja pallida* venom and ceftriaxone (*p*=0.2368), *Naja pallida* venom and nalidixic acid (*p*=0.9688), *Naja pallida* venom and tetracycline (*p* > 0.9999), and *Naja pallida* venom and sulfamethoxazole (*p*=0.9688) as shown in Figure 7.

Table 2 is a summary of the minimum inhibitory concentration of *Naja haje* venom against some Gram-positive and Gram-negative bacteria.

## 4. Discussion

In 2017, the World Health Organization published a list of antibiotic resistant bacteria. This list was dubbed as the “WHO priority pathogen list.” It is divided into three key priorities based on the urgency and need for new antibiotics, i.e., priority 1: critical, priority 2: high, and priority 3: medium.

In the present study, we evaluated the antimicrobial activities of *Bitis arietans, Naja haje,* and *Naja pallida* against Gram-positive (*B. cereus, S. aureus,* and *S. typhi*) and Gram-negative (*E. coli* and *K. pneumoniae*). Organisms such as *E. coli* and *K. pneumoniae* are WHO priority 1 (critical) pathogens while organisms such as *S. typhi* and *S. aureus* are WHO priority 2 (high) pathogens.

The clinical and laboratory standard institute (CLSI) has developed zone diameter and minimum inhibitory concentration breakpoints of various antibiotics when tested against various pathogens [17]. Based on these breakpoints, it is possible to determine whether the pathogens are sensitive, intermediate, or resistant to a test substance/compound [17]. When these criteria are considered, *E. coli* was found to be sensitive to aztreonam, cefpodoxime, cefoxitin, ceftriaxone, nalidixic acid, and tetracycline. *K. pneumoniae* was sensitive to streptomycin and tetracycline but resistant to sulfamethoxazole, aztreonam, and cefpodoxime. *S. aureus* was sensitive to cefoxitin, streptomycin, ceftriaxone, and tetracycline but resistant to cefpodoxime, nalidixic acid, and sulfamethoxazole. *S. typhi* was sensitive to cefoxitin and ceftriaxone but resistant to aztreonam, sulfamethoxazole, nalidixic acid, streptomycin, and tetracycline.

Culture samples from fangs, fang sheaths, and the oral cavities of venomous snakes have been shown to have an array of microbes such as *Bacillus subtilis, Morganelli morganii,* and coagulase-negative *Staphylococci.* Others include *Pseudonomas, Staphylococcus, Salmonella, Streptococci, Enterobacter, Escherichia, Citrobacter, Proteus,* and *Clostridium sp* which are all potentially pathogenic [18–21]. It is expected that snake mouth bacteria may be inoculated during a dry bite or envenomation resulting in infection. However, it is fascinating that snakebite victims rarely suffer from complications arising from bacterial infections [21]. One of the first reports to evaluate the antimicrobial properties of snake venom was by Glaser in 1948 [22]. Since then, the field has grown exponentially to the point that individual snake venom proteins are being explored for antimicrobial activity [23–27].

The results of the present study suggest that both Gram-positive and Gram-negative bacteria were resistant to *B. arietans* venom as no inhibition was observed in the antibacterial assay. These results are contrary to those of Al-Asmari and colleagues who reported that *Bitis arietans* venom from captive bred snakes in Saudi Arabia was effective against *S. aureus, E. fecalis*, and *P. aeruginosa* [28].

Elapid venoms (e.g., *Naja haje* and *Naja pallida* venoms) have been reported to have significantly higher percentages of three finger toxins (3FTx's) than viperid venoms (*Bitis arietans* venom) [29, 30]. The three finger toxins (3FTx's) have been reported to have higher specific activity towards the lipids contained within the Gram-positive plasma membranes than those found in Gram-negative bacterial membranes [31]. Not unexpectedly, the elapid venoms studied were more effective than at inhibiting Gram-positive bacteria than Gram-negative bacteria. Similar observations were made by Charvat and colleagues [32].

The exact mechanism of antimicrobial activity of 3FTxs is not known. However, it is postulated that 3FTxs cause membrane destabilization and release of cytoplasmic materials in bacteria [32–34]. L-amino acid oxidases (LAAOs) in venoms have also been implicated in morphological alterations in bacteria including disruption of the mitochondrial membranes leading to total destruction and/or loss of organelles [35, 36].

## 5. Conclusions

In conclusion, these findings suggest that *Naja haje* and *Naja pallida* venoms have better antibacterial activity than some of the antibiotics which are currently in use for microbial infections. However, the venom of *Bitis arietans* appears to be ineffective against common bacterial pathogens.

### 5.1. Limitations

A limited number of venoms (from one viper, one spitting cobra, and one nonspitting cobra) was used in this study. Future studies should employ a broader range of snake venoms including those from the Mambas and colubrids found in Subsaharan Africa.

## Figures and Tables

**Table 1:** Antibacterial effect of conventional antibiotics and venoms against gram-positive and gram-negative bacteria.

Microorganism	Inhibition zone (mm)^a^										
	*BAV*	*NHV*	*NPV*	ATM	CPD	CXT	STM	CRO	NAL	TCY	SMZ
*E. coli*	0.0 ± 0.0	7.6 ± 0.5	6.3 ± 0.8	28.6 ± 2.2	25.1 ± 0.9	22.6 ± 1.1	14.0 ± 0.4	28.2 ± 0.6	21.7 ± 0.7	25.8 ± 1.0	15.9 ± 0.2
*K. pneumoniae*	0.0 ± 0.0	6.8 ± 0.2	5.8 ± 0.7	14.0 ± 0.2	12.6 ± 0.2	17.4 ± 0.3	15.3 ± 1.8	19.9 ± 0.6	15.6 ± 0.3	20.8 ± 0.3	0.0 ± 0.0
*S. aureus*	0.0 ± 0.0	12.1 ± 1.7	9.7 ± 0.5	0.0 ± 0.0	0.0 ± 0.0	22.5 ± 0.7	29.4 ± 1.8	25.0 ± 3.0	0.0 ± 0.0	26.9 ± 0.7	0.0 ± 0.0
*B. cereus*	0.0 ± 0.0	9.8 ± 0.7	10.8 ± 0.7	0.0 ± 0.0	0.0 ± 0.0	25.2 ± 0.6	33.0 ± 1.7	14.6 ± 0.5	0.0 ± 0.0	32.8 ± 0.8	25.0 ± 2.2
*S. typhi*	0.0 ± 0.0	7.7 ± 1.7	6.2 ± 0.5	0.0 ± 0.0	20.7 ± 0.6	20.0 ± 0.1	10.2 ± 0.4	29.8 ± 0.4	0.0 ± 0.0	9.3 ± 0.2	0.0 ± 0.0

^a^The values represent inhibition zone in millimeters, after 24 h incubation performed in triplicate assays. BAV: *Bitis arietans* venom; NHV; *Naja haje* venom; NPV: *Naja pallida* venom; ATM: aztreonam; CPD: cefpodoxime; CXT: cefoxitin; STM: streptomycin; CRO: ceftriaxone; NAL: nalidixic acid; TCY: tetracycline; SMZ: sulfamethoxazole.

**Table 2:** Minimum inhibitory concentration of *Naja haje* venom against some gram positive and gram negative bacteria.

Microorganism	MIC (mg/mL)
*B. cereus*	1.72 ± 0.00
*E. coli*	0.96 ± 0.45
*K. pneumoniae*	1.33 ± 0.00
*S. aureus*	1.25 ± 0.00
*S. typhi*	1.03 ± 0.55

## Data Availability

The datasets used and/or analyzed during this study are available from the corresponding author on reasonable request.
